# Characteristics, management and outcomes of primary hyperparathyroidism from 2009 to 2021: a single centre report from South Africa

**DOI:** 10.1186/s12902-024-01583-8

**Published:** 2024-04-25

**Authors:** Kamal Govind, Imran M. Paruk, Ayesha A. Motala

**Affiliations:** Department of Diabetes and Endocrinology, University of KwaZulu-Natal, Durban, South Africa

**Keywords:** Primary hyperparathyroidism, Hypercalcaemia, Parathyroid hormone, Hungry bone syndrome, Parathyroidectomy, Intra-operative parathyroid hormone monitoring

## Abstract

**Background:**

There has been a notable shift towards the diagnosis of less severe and asymptomatic primary hyperparathyroidism (PHPT) in developed countries. However, there is a paucity of recent data from sub-Saharan Africa (SSA), and also, no reported data from SSA on the utility of intra-operative parathyroid hormone (IO-PTH) monitoring. In an earlier study from Inkosi Albert Luthuli Central Hospital (IALCH), Durban, South Africa (2003–2009), majority of patients (92.9%) had symptomatic disease. The aim of this study was to evaluate the clinical profile and management outcomes of patients presenting with PHPT at IALCH.

**Methods:**

A retrospective chart review of patients with PHPT attending the Endocrinology clinic at IALCH between July 2009 and December 2021. Clinical presentation, laboratory results, radiologic findings, surgical notes and histology were recorded.

**Results:**

Analysis included 110 patients (87% female) with PHPT. Median age at presentation was 57 (44; 67.5) years. Symptomatic disease was present in 62.7% (n:69); 20.9% (n:23) had a history of nephrolithiasis and 7.3% (n:8) presented with previous fragility fractures. Mean serum calcium was 2.87 ± 0.34 mmol/l; median serum-PTH was 23.3 (15.59; 45.38) pmol/l, alkaline phosphatase 117.5 (89; 145.5) U/l and 25-hydroxyvitamin-D 42.9 (33.26; 62.92) nmol/l. Sestamibi scan (n:106 patients) identified an adenoma in 83.02%. Parathyroidectomy was performed on 84 patients with a cure rate of 95.2%. Reasons for conservative management (n:26) included: no current surgical indication (n:7), refusal (n:5) or deferral of surgery (n:5), loss to follow-up (n:5) and assessed as high anaesthetic risk (n:4). IO-PTH measurements performed on 28 patients indicated surgical success in 100%, based on Miami criteria. Histology confirmed adenoma in 88.1%, hyperplasia in 7.1% and carcinoma in 4.8%. Post-operative hypocalcaemia developed in 30 patients (35.7%), of whom, 14 developed hungry bone syndrome (HBS). In multivariate analysis, significant risk factors associated with HBS included male sex (OR 7.01; 95% CI 1.28, 38.39; p 0.025) and elevated pre-operative PTH (OR 1.01; 95% CI 1.00, 1.02; p 0.008).

**Conclusions:**

The proportion of asymptomatic PHPT has increased at this centre over the past decade but symptomatic disease remains the dominant presentation. Parathyroidectomy is curative in the majority of patients. IO-PTH monitoring is valuable in ensuring successful surgery.

## Introduction

Primary Hyperparathyroidism (PHPT) is a disorder of calcium homeostasis defined by hypercalcaemia and elevated or inappropriately normal levels of parathyroid hormone (PTH) [1–3]. From available reports, there is a high incidence in the western world, as high as 8.2/1000 person-years in the United States of America (USA) with a female preponderance (F: M; 3–4:1) [1]. Solitary benign parathyroid adenoma accounts for the majority (± 80%) of PHPT, with multiglandular disease accounting for 15–20% [1, 3, 4].

Symptomatic PHPT presents with a multitude of complications including skeletal, renal, neuropsychiatric and cardiovascular manifestations, classically described as “bones, stones, groans and moans” [1, 3, 4]. The clinical presentation in developed countries has evolved from one of symptomatic severe disease to asymptomatic and often incidentally discovered [1, 3]. Earlier diagnosis through adoption of routine calcium testing in the 1970’s [2] and an increase in screening for osteoporosis in the late 1990’s [3], may account for the lower frequency of symptomatic disease. As routine tests are also increasingly available in developing countries, there has been a shift in the spectrum towards less severe and asymptomatic disease, as reported in recent studies from India and China [5, 6].

Vitamin D deficiency is frequently found in patients with PHPT, with a reported incidence of 91–100% [7]. The relationship between vitamin D deficiency and PHPT remains unclear. Although vitamin D deficiency is usually associated with secondary hyperparathyroidism, it has been postulated that vitamin D deficiency may lead to parathyroid hyperplasia or adenomatous change and possibly greater disease severity in PHPT [3, 5, 7, 8].

Despite the changes in disease presentation, parathyroidectomy is still the only cure for PHPT, especially for symptomatic patients [3]. The use of intraoperative parathyroid hormone (IO-PTH) monitoring during parathyroidectomy has become valuable in assisting surgeons to ascertain if all hyperfunctioning parathyroid tissue was excised. IO-PTH has been shown to be highly sensitive and specific in predicting operative success [9].

There is a paucity of data on patients with PHPT from Africa, and only two previous reports from South Africa [10, 11]. In a study from our centre (Inkosi Albert Luthuli Central Hospital (IALCH)) in Durban, the characteristics and outcome of PHPT were analysed in 28 patients over 6 years (2003–2009). The majority (92.9%) of patients had symptomatic disease and surgery was successful in 94.7% of patients [10]. A more recent report from Cape Town also reviewed BMD findings, rates of vertebral fractures and osteitis fibrosa cystica in patients with PHPT who had parathyroidectomy; 50% of post-menopausal women and older men had osteoporosis and 21% of patients had vertebral fractures [11]. The limited information highlights the need for further reports from South Africa and sub-Saharan Africa (SSA) regarding the frequency of asymptomatic disease presentation, vitamin D status of patients with PHPT and the utility of intra-operative PTH (IO-PTH) monitoring.

This study was undertaken to determine the clinical, laboratory and radiologic features and outcomes of management of patients presenting with PHPT between 2009 and 2021; also to determine whether there was a change over the past decade.

## Research design and methods

### Study design and study population

This was a retrospective chart review of all patients with primary hyperparathyroidism referred to the adult endocrinology clinic at IALCH from July 2009 to December 2021. Patients with familial hypocalciuric hypercalcaemia, tertiary hyperparathyroidism, lithium-related hypercalcaemia, diuretic-associated hypercalcaemia and those patients under 12 years of age were excluded.

### Data collection

The files of all patients with PHPT were accessed on the electronic health record (EHR) system at IALCH. Data recorded for each patient included demographic details, symptomatology, past medical history and clinical parameters. Pre-operative laboratory results recorded included: serum PTH, corrected calcium, phosphate, magnesium, urea, creatinine, alkaline phosphatase (ALP), 25-hydroxyvitamin D, 1,25-hydroxyvitamin D, lipid profile and HbA_1C_. Estimated glomerular filtration rate (eGFR) was calculated using the Chronic Kidney Disease Epidemiology Collaboration (CKD-EPI) equation [12]. Twenty-four hour urine calcium excretion and spot urine calcium to creatinine excretion ratio were also recorded. Serum PTH was analysed using chemiluminescence immunoassay (normal reference range: 2.0-8.5 pmol/l).

### Definitions

Where IO-PTH monitoring was undertaken, blood was drawn before and 10 minutes after resection of the adenomas. Surgical success was defined using the Miami criteria, i.e. a > 50% drop in PTH levels at 10 minutes after the excision of all hyperfunctioning parathyroid gland(s) [9, 13]. Vitamin D deficiency was defined as a serum 25-OH-Vitamin D level of < 30nmol/l. Hungry bone syndrome (HBS) was defined as profound (corrected serum calcium < 2.1 mmol/l) and prolonged (longer than four days post-operatively) hypocalcaemia following parathyroidectomy [14]. Osteoporosis was defined as BMD T-score > -2.5 below the young adult mean or Z-score > -2.5 below the mean for males < 50 years and premenopausal females.

Pre-operative radiological data recorded included skeletal survey, bone mineral density (BMD) scan using dual-energy X-ray absorptiometry (DEXA), renal ultrasound and technetium (Tc-99 m) sestamibi scan. Findings of parathyroid ultrasound were not recorded as it was only introduced at IALCH recently. Surgical notes were reviewed to note if a culprit lesion was identified at surgery and histology reports were reviewed for a final diagnosis (parathyroid adenoma, hyperplasia or carcinoma).

### Post-operative evaluation

Laboratory results recorded within the first 24 hours post-operatively included serum PTH, corrected calcium, phosphate, magnesium and ALP. The corrected calcium, phosphate and magnesium were recorded daily for up to 6 days/until time of discharge as part of evaluation for post-operative hypocalcaemia and/or HBS. The results at discharge were also recorded and used in the final analyses. Where available, repeat BMD results during outpatient follow-up were documented with areal BMD (aBMD) values used to monitor changes post-operatively.

### Statistical analysis

Statistical analysis was performed using SPSS (Statistics Package for the Social Sciences) version 28. Data are presented as mean ± SD, median (Interquartile Range - IQR) or %. For univariate and bivariate analysis, continuous variables were compared using the Student’s t-test and categorical variables compared with Pearson′s Chi-Square (Χ^2^) test. Multivariate analysis for predictors (risk factors) for HBS was undertaken using logistic regression and reported as OR(95% CI)p. The performance of sestamibi scan in predicting successful resection of the culprit lesion was assessed by correlating sestamibi scan results with surgeon’s finding at surgery, by cross-tabulation. A p value of < 0.05 was considered statistically significant.

## Results

### Demography and clinical presentation

The study population included 110 patients, with median age 57 (44; 66.8) years; 87.3% were female and the majority were of African or Indian ethnicity (Table 1). The median BMI was 27.4 (23.39; 32.85) kg/m^2^ and median blood pressure 130 (116; 146) mmHg systolic and 75 (66; 82) mmHg diastolic.

**Table 1:** Clinical and biochemical characteristics of the total study group at presentation (n: 110)

	N	%
Sex (M : F)	14 : 96	12.7 : 87.3
Ethnicity		
Indian	48	43.6
African	45	40.9
White	16	14.5
Mixed Race (Coloured)	1	1
		Range
Age (years)	57 (44; 66.8)	13–84
BMI (kg/m^2^)	27.4 (23.4; 32.9)	14.55–49.98
Systolic Blood Pressure (mmHg)	130 (116; 146)	92–186
Diastolic Blood Pressure (mmHg)	75 (66; 82)	46–104
	N	%
**Past History Attributable To PHPT**	43	39.1
Nephrolithiasis	23	20.9
Non-trauma fracture	8	7.3
Osteoporosis	7	6.4
Peptic Ulcer Disease	6	5.5
Depression	3	2.7
Pancreatitis	2	2
Psychosis	1	1
Other Medical History	N	%
Hypertension	66	60.5
Diabetes Mellitus	29	26.4
Dyslipidaemia	31	28.2
Malignancy	10	9.1
Baseline Biochemistry		Normal Reference Range
Serum Calcium (mmol/l)	2.87 ± 0.34	2.15–2.50
Serum Magnesium (mmol/l)	0.82 ± 0.12	0.63–1.05
Serum Phosphate (mmol/l)	0.87 ± 0.20	0.78–1.42
Serum Creatinine (µmol/l)	71 (58; 95)	49–90
eGFR (CKD-EPI) (ml/min/1.73m^2^)	85.9 ± 33.4	> 60
Serum ALP (U/L)	117.5 (89; 145.5)	42–98
Serum PTH (pmol/l)	23.3 (16.0; 45.4)	2.0-8.5
*Serum 25-OH Vitamin D (nmol/l) (n:95)	42.9 (33.3; 62.9)	> 50
Vitamin D deficiency (n;%)	16; 16.8	
24-hour urine calcium (mmol/day)	3.3 (1.6; 5.4)	2.5–7.5

Data are presented as Mean ± SD or Median (IQR) or %

BMI: body mass index

Vitamin D Deficiency: serum 25-OH-vitamin D < 30 nmol/l

* Serum 25-OH Vitamin D available in 95 patients

**Fig. 1:**
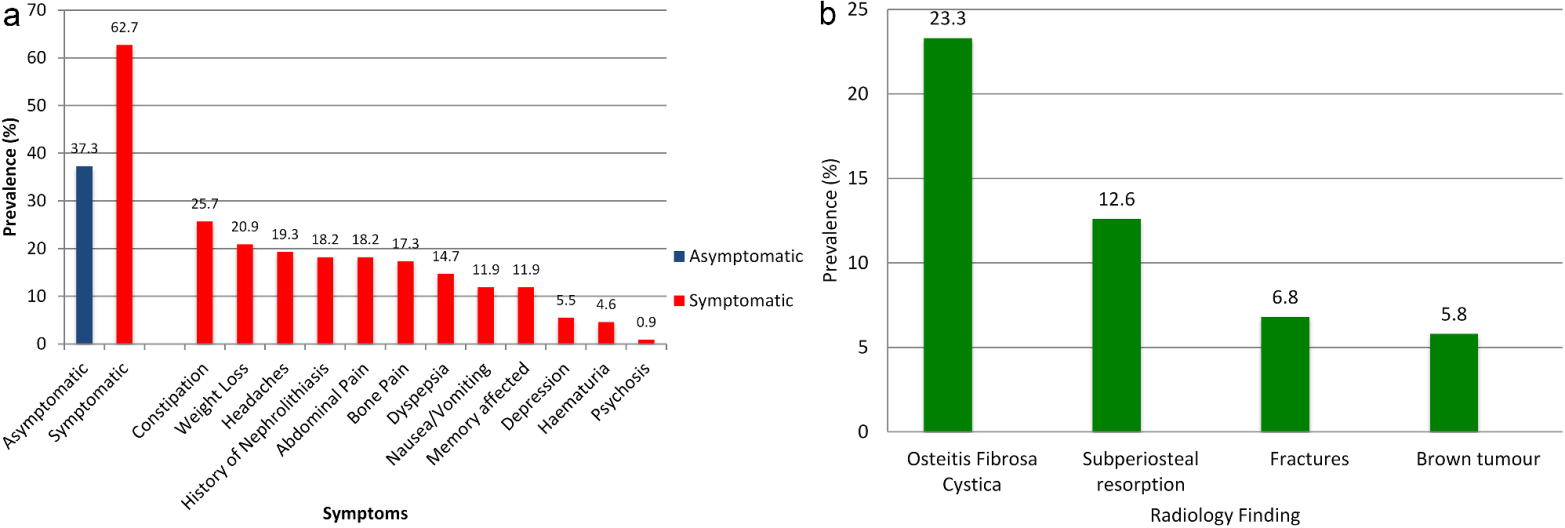
Prevalence of symptoms at presentation (n:110)(Fig. 1a), and radiological abnormalities on plain x-rays at presentation (n:103)(Fig. 1b)

A past history suggestive of PHPT was reported in 39.1% (n:43) with nephrolithiasis (n: 23) the most frequent. In 10 patients with a history of malignancy, 4 patients had previously treated breast cancer (Table 1). At presentation, 37.3% (n:41) were asymptomatic while 62.7% (n:69) were symptomatic; the most common symptoms were constipation (25.7%) and weight loss (20.9%) (Fig. 1a).

### Baseline biochemistry and radiology

Table 1 shows the baseline biochemistry results. Mean serum calcium was 2.87 ± 0.34 mmol/l and mean phosphate 0.87 ± 0.12 mmol/l; median ALP 117.5 (89; 145.5) U/L and median PTH 23.3 (15.95; 45.38) pmol/l. Median 24-hour urine calcium excretion was 3.33 (1.58; 5.41) mmol/24 hours. Median 25-hydroxyvitamin D was 42.9 (33.26; 62.92) nmol/l.

The most frequently reported abnormality on x-ray was osteitis fibrosa cystica (23.3%) (Fig. 1b). Osteoporosis was found in 54.9% (51/93) on BMD with mean T-score - 2.74 ± 1.83 and mean Z-score - 1.65 ± 2.01 at distal 1/3 of radius. In 106 patients who had Sestamibi scans, 83% (n:88) had evidence of a parathyroid adenoma; the remainder were negative.

### Management

Surgery was undertaken in the majority (76.4%; n:84) of patients; 26 patients were managed conservatively, for the following reasons: no current surgical indication (n:7), refusal (n:5) or deferral of surgery (n:5), loss to follow-up (n:5) and deemed a high anaesthetic risk (n:4). Of the surgical group, 40% (n:34) received intravenous bisphosphonates pre-operatively, while none received cinacalcet or calcitonin.

Operative and Post-operative:

At surgery, a resectable lesion was identified in 95.2% (n:80) (80/84). On histology, the majority (n:73) of patients were confirmed to have single parathyroid adenoma; one patient had 2 adenomas. 7.1% (n:6) had hyperplasia and 4.8% (n:4) had parathyroid carcinoma (Table 2). The surgeon’s macroscopic finding of an adenoma correlated with histology in 91.3% (73/80) of patients; in 3 (3.8%) patients, histology showed hyperplasia and in 4 (5%) carcinoma. Three of the four patients with parathyroid carcinoma had en-bloc parathyroidectomy with ipsilateral thyroid lobectomy; one patient initially had a focused parathyroidectomy only, but subsequently underwent thyroid lobectomy and cervical lymph node dissection after initial excision of the affected parathyroid gland was incomplete on histopathological analysis. Of the 4 patients with no resectable lesion identified during surgery, histology from sub-total parathyroidectomy confirmed a single adenoma in 1 patient and hyperplasia in 3 patients. Data on adenoma sizes and weights were not consistently available and could not be analysed.

**Table 2:** Outcomes of surgery, intra-operative parathyroid hormone (IO-PTH) monitoring and histopathology (n:84)

	n	%
Parathyroidectomy	84	100
Cure	80	95.2
No cure	4	4.8
IO-PTH Monitoring		
IO-PTH	28	33.3
Success/Cure	28	100
No IO-PTH	56	66.7
Success/Cure	52	92.9
Histological Diagnosis		
Adenoma	74	88.1
Hyperplasia	6	7.1
Carcinoma	4	4.8

IO-PTH: Intra-operative parathyroid hormone monitoring

At discharge post-surgery, there was a reduction in median serum calcium from 2.90 (2.67; 3.13) to 2.25 (2.15; 2.35) mmol/l and PTH from 24.65 (16.78; 56.58) to 4.45 (2.1; 8.75) pmol/l with increase in mean serum phosphate from 0.83 ± 0.196 to 1.15 ± 0.68 mmol/l.

Post-operative hypocalcaemia developed in 35.7% (30/84) of patients; 16.6% (n:14) developed hungry bone syndrome (HBS), requiring intravenous calcium supplementation. 10 of the 14 patients who developed HBS received intravenous bisphosphonates prior to parathyroidectomy. Hypocalcaemia was transient in 20.2% (n:17) and permanent in 15.5% (n:13). Most patients with post-operative hypocalcaemia (n:19) were on oral calcium replacement at discharge; 13 patients (43.3%) required additional one-alpha calcidiol therapy (Table 3).

**Table 3:** Frequency of post-operative complications and replacement therapy

	n	%
Complication	84	
Hypocalcaemia	30	35.7
Transient	17	20.2
Permanent	13	15.5
Hungry Bone Syndrome	14	16.6
Recurrent laryngeal nerve injury	0	0
Bleeding	0	0
Infection	0	0
Treatment for Hypocalcaemia	n:30	35.7
Intravenous Calcium	14	46.7
Oral Calcium	19	63.3
One-Alpha Calcidiol	13	43.3
No replacement	11	36.7

Post-operative BMD done on 20 patients (median follow-up 15.5 months) showed significant improvement only at the spine (+ 6.5%).

The surgical cure rate was 95.2% (n: 80) (Table 2). Reasons for failed surgery (n:4) included persistent PHPT due to incomplete excision (n:2) and recurrent disease with evidence of a new adenoma at follow-up 1 year after first surgery (n:2).

On bivariate analysis, when compared with patients who did not develop HBS, patients with HBS were significantly younger and leaner, had higher serum PTH and ALP and lower phosphate at presentation; eGFR was higher and HbA_1C_ lower. Mean BMD was lower at all sites (Table 4). In multivariate analysis using logistic regression, significant predictive risk factors for HBS in this cohort included male sex (OR 7.01; 95% CI 1.28, 38.39; p 0.025) and pre-operative serum PTH (OR 1.008; 95% CI 1.00, 1.02; p 0.008) (Table 5).

**Table 4:** Characteristics at presentation in patients with and without Hungry Bone Syndrome (HBS) (n:84)

	Pre-operative		
	HBS (n:14)	No HBS (n:70)	P
Age (years)	39.5 (20.8; 56)	58 (47; 67)	0.012
BMI (kg/m^2^)	22.7 (17.8; 24.1)	27.4 (23.7; 32.4)	0.005
Serum:			
PTH (pmol/l)	172.7 (71.2; 230)	23.1 (15.95; 38.9)	< 0.00
ALP (U/L)	900 (174.8; 1540.3)	117 (88; 140.3)	0.000
Calcium (mmol/l)	3.06 ± 0.45	2.88 ± 0.30	0.084
Magnesium (mmol/l)	0.78 ± 0.13	0.82 ± 0.11	0.304
Phosphate (mmol/l)	0.73 ± 0.16	0.87 ± 0.19	0.018
aBMD Hip (g.cm^2^)	0.6 ± 0.3	0.8 ± 0.2	0.015
aBMD Spine (g.cm^2^)	0.7 ± 0.2	0.8 ± 0.2	0.013
aBMD Radius (g.cm^2^)	0.4 ± 0.1	0.6 ± 0.1	0.002
eGFR (CKD-EPI) (ml/min/1.73m^2^)	114 ± 39.1	83.5 ± 30.5	0.002
HbA1c (%)	5.4 (5.3; 5.8)	6.3 (5.8; 7.9)	0.008

Data are presented as Mean ± SD or Median (IQR).

BMI: Body Mass Index; PTH: Parathyroid Hormone; ALP: Alkaline phosphatase

**Table 5:** Logistic regression analysis for predictors (risk factors) for hungry bone syndrome (HBS)

Variable	OR (95% C.I)	P
PTH	1.0 (1.0, 1.02)	0.008
Male Gender	7.0 (1.3, 38.4)	0.025
24-hour urine calcium	1.2 (1.0, 1.5)	0.107
Phosphate	0.02 (0.0, 2.8)	0.239

PTH: Parathyroid Hormone

OR (95% C.I.): Odds Ratio (95% confidence interval)

Compared to patients without Vitamin D deficiency, patients with Vitamin D deficiency (n: 16) were significantly younger (42 vs. 59 years)(*p* = 0.008), and had higher median pre-operative calcium (2.95 vs. 2.83 mmol/l)(*p* = 0.029) and ALP (202 vs. 103 U/l)(*p* = 0.00); median BMD T-scores were lower at the hip (-2.0 vs. -1.1)(*p* = 0.033), spine (-3.3 vs. -1.8)(*p* = 0.013) and radius (-4.5 vs. -2.4)(*p* = 0.006) (Table 6). In bivariate analysis, significant predictors for Vitamin D deficiency were non-trauma or fragility fractures (*p* = 0.032) and osteitis fibrosa cystica (*p* < 0.001).

**Table 6:** Comparison of clinical parameters, biochemistry and cure rate between 2009 (n:28) and current (n:110) study

	2009	2021	P
	n:28	n:110	
Symptomatic (%)	92.9	62.7	0.002
BMI (kg/m^2^)	28.1 (26.1; 31.1)	27.3 (23.4; 32.9)	0.462
Pre-operative			
Serum Calcium (mmol/l)	3.00 (2.9; 3.2)	2.83 (2.6; 3.1)	0.010
Serum PTH (pmol/l)	16.85 (9.9; 40.7)	23.3 (16.0; 45.4)	0.824
25-OH Vit D (nmol/l)	39 (26; 47.9)	42.9 (33.3; 62.9)	0.488
Serum ALP (U/l)	98 (79; 125)	117.5 (89; 145.5)	0.133
24-hour urine calcium (mmol/day)	5.1 (2.5; 6.1)	3.34 (1.59; 5.4)	0.654
Post-operative			
Serum PTH (pmol/l)	2.3 (1.1; 4.5)	4.4 (2.1; 8.8)	0.044
Serum Calcium (mmol/l)	2.31 (2.2; 2.4)	2.25 (2.15; 2.4)	0.233
Cure (%)	94.7	95.2	0.819

Data are presented as Median (IQR) or %

BMI: body mass index; PTH: Parathyroid Hormone, 25-OH Vit D: 25-hydroxyvitamin D; ALP: Alkaline phosphatase

Preoperative sestamibi scans (in 82 of 84 patients) had a sensitivity of 93.9%, specificity of 100% and positive predictive value of 100% for successful location and resection of the culprit lesion.

There was a 100% cure rate in the 28 patients who had IO-PTH monitoring, while patients who did not have IO-PTH monitoring had a 92.9% cure rate (Table 2).

When compared with our previous report in 2009 [10], there was a significantly higher frequency of asymptomatic presentation of PHPT (37.3 vs. 7.1%) [*p* = 0 0,003].

## Discussion

In this study, there was a higher proportion of asymptomatic PHPT compared to the previous study at the same centre, but the prevalence of symptomatic disease is still high and parathyroid adenoma remains the major cause of PHPT. IO-PTH monitoring is valuable in confirming successful surgery.

The increase in asymptomatic disease from 7.1% reported in our previous study in 2009 [10] to 37.3% in the present study, although lower than the predominantly asymptomatic PHPT found in developed countries [1, 3], is similar to that observed in other developing countries such as China and India [15, 16]. This can be explained by wider implementation of routine serum calcium measurement as part of biochemical screening, even in developing countries.

The finding that the major cause of PHPT in this study was a solitary benign parathyroid adenoma is compatible with most other studies reported globally [1, 3–6]. Parathyroid carcinoma accounted for 4.8% of histological diagnoses in this group. This is similar to rates reported in India and China [5, 6].

In this study, IO-PTH confirmed surgical success of focused parathyroidectomy in all cases (100%) and highlights the accuracy of the Miami criterion for predicting surgical cure. This is higher than that reported in other studies (97–98%) [9, 17] and may largely be explained by having a highly experienced, dedicated parathyroid surgeon. However, IO-PTH was only done in one third of the surgical group, highlighting the need to confirm this in a larger group and to evaluate emerging alternate criteria [9, 17–19].

As reported in other studies [16, 20–22], nephrolithiasis was the most common presenting feature; the prevalence of osteoporosis was lower compared with studies reporting similar rates of symptomatic PHPT [5, 6, 16, 21, 22] and may be explained by less frequent screening for hypercalcaemia at primary levels of care.

As in other studies, hypercalcaemia was present in the majority of patients [15]. The proportion (15.6%) with normocalcaemic PHPT (NPHPT) is higher than the reported prevalence of 0.1–8.9% [23]. These may be patients with mild PHPT or alternatively a variant of PHPT that has variable rates of progression to hypercalcaemia and is unlikely due to Vitamin D deficiency [24, 25].

Vitamin D deficiency was found in 16.8% (n:16/95) of patients in this study; this is similar to rates reported in both developed and developing countries [26, 27]. Patients with vitamin D deficiency were younger, had higher pre-operative calcium, higher ALP levels and lower median BMD scores at all sites than those without Vitamin D deficiency. Vitamin D deficiency has been associated with severe disease [8, 28] and these findings could indicate that it may be associated with greater skeletal disease burden and, thus, lower BMD. However, the low prevalence of Vitamin D deficiency makes it difficult to draw such conclusions.

The high rate of surgical cure in this study (95.2%) is comparable to rates in other studies with highly skilled, dedicated surgeons using minimally-invasive techniques guided by preoperative localization imaging [29, 30]. In our centre, preoperative localization with Tc-99 sestamibi was highly sensitive and specific in predicting successful resection of the culprit lesion and is consistent with findings in developed as well as other developing countries such as India [31, 32]. Although the sensitivity of parathyroid ultrasound for adenoma detection has generally improved in other countries as a result of improving sonographic technology [33, 34], it could not be evaluated in this study as parathyroid ultrasound was only introduced at our centre in the last 2 years.

Post-operatively, HBS developed in 16.6% of patients, which is similar to other reports from Europe and the Middle East [35–37], but higher than rates observed in a recent report from Singapore [38]. The effects of preoperative bisphosphonate therapy are unclear, however, several reports have suggested that it may prevent or reduce HBS [14, 39–42]. This effect may likely be related to the reduction in bone-turnover effected by bisphosphonates prior to surgery [14]. Significant predictors for HBS included male sex and a higher pre-operative PTH level, which were also found in recent reports [35–37]. Identifaction of patients with these risk factors may enable prompt perioperative treatment and decrease morbidity and hospitalisation.

The observation that post-operative increase in BMD was only significant at the spine is in keeping with results of recent studies from China with similar follow-up intervals [43, 44]. This may be explained by rates of bone turnover being higher in cancellous bone, resulting in rapid and lasting improvement in BMD [43, 44]. However, since less than 24% of patients had repeat BMD measurements post-operatively, the results need to be interpreted with caution and requires further investigation.

Comparison with the earlier study from this centre showed that patients’ clinical characteristics, biochemical findings and cure rate are largely similar. This highlights the need for increased routine biochemical testing at primary care centres in order to detect more patients with asymptomatic PHPT.

The strength of this study is that it is the largest clinical study done on PHPT in South Africa and that it can be compared to the previous study at our centre. There are several limitations including the retrospective nature, lack of data on adenoma size and weight, limited number of patients who had IO-PTH monitoring, and follow-up BMD measurements post-surgery. Further studies are needed in other centres in South Africa and other African countries to confirm the findings of this study, ascertain trends in BMD changes post-operatively as well as assess the utility and cost-effectiveness of IO-PTH monitoring.

## Conclusion

The proportion of asymptomatic PHPT has increased at this centre over the past decade but symptomatic disease remains the dominant presentation and is higher than that reported in developed countries. Parathyroid adenoma is the commonest cause of PHPT with parathyroidectomy being curative in the majority of patients. IO-PTH monitoring is valuable in guaranteeing successful surgery.

## Data Availability

The datasets generated and/or analysed during the current study are not publicly available due to patient confidentialty, but are available from the corresponding author on reasonable request.

## Abbreviations

PHPT: Primary Hyperparathyroidism
SSA: Sub-Saharan Africa
IO-PTH: Intraoperative parathyroid hormone
IALCH: Inkosi Albert Luthuli Central Hospital
PTH: Parathyroid hormone
USA: United States of America
EHR: Electronic health record
ALP: Alkaline phosphatase
eGFR: Estimated glomerular filtration rate
CKD-EPI: Chronic Kidney Disease Epidemiology Collaboration
HBS: Hungry Bone Syndrome
BMD: Bone Mineral Density
DEXA: Dual-Energy X-ray Absorptiometry
Tc-99m: Technetium
SPSS: Statistics Package for the Social Sciences
IQR: Interquartile range
OR: Odds Ratio
95% CI: 95% confidence interval
BMI: Body mass index
NPHPT: Normocalcaemic primary hyperparathyroidism
